# Clinical Reflections

**Published:** 1882-01

**Authors:** Ad. Lippe

**Affiliations:** Philadelphia


### CLINICAL REFLECTIONS.

AD. LIPPE, M. D., PHILADELPHIA.

On the morning of the 2d of November, I was summoned to see Mrs. C., æt. fifty years. In early life this lady resided on the banks of the Delaware, and with the other residents in that locality, suffered very severely from intermittent fever, and still more from enormous doses of Quinine, administered year after year for the suppression of that disease.

For more than twenty years she has been under homœopathic treatment, and the admonitory symptoms of chills and fever returned gradually with less severity every spring and autumn. For the last ten years they were very light, only occasionally reminding her of her previous sufferings; her brain and spleen which had been injured severely from former overdoses were now in an almost normal condition. Early in October, while on a visit to West Point, on the Hudson river, she was exposed to the then prevailing malarial influences, and began to feel badly. Returning to Philadelphia, she suffered, first, from diminished appetite, followed by frequently returning watery, but painless diarrhœa, for which she took no remedy, hoping that the disease would be carried off in that way. On the night of the 1st of November, she felt very cold on retiring to bed, and was awakened about 2 A. M., by a very heavy chill which made her teeth chatter; she had to rise, feeling nausea and a desire to have a stool, and went to the water-closet back of the back chamber. After a very profuse watery evacuation, she tried to walk to the front chamber, but on reaching the door, was overcome by weakness and fell to the floor. She chattered and shook all over from the chill, became stiff and icy cold to the touch, and, in an unconscious state, was carried to bed. Heat was applied and when consiousness returned she was totally blind, not being able to see the strongest gaslight. About 4 A. M., fever set in and she fell into a dose, her breathing very much oppressed. When I saw her at 9 A. M., she was suffering from extreme prostration; was not aware of her unconsciousness at night but remembered the blindness. She complained that the external and icy coldness was not to be compared with the icy coldness she had felt about the heart, that even then she felt cold and pain about the heart, that she had slight headache and backache, and no perspiration after the slight fever heat. The pulse was very weak, irregular, small and less than fifty beats in a minute. She had a dry mouth but no thirst, aversion to food, and her position in bed clearly showed her intense debility, of which she complained bitterly. She received one dose of *Natrum mur.* CM (Fk.) dry on the tongue. When I saw her at 7 P. M., November 2d, she had perspired slightly during the day, the tongue and mouth felt less dry, no thirst, pulse over sixty beats in a minute and more regular. November 3d, she was feeling better, and her condition continued to improve without further medication. On the 6th of November, I dined with her at 6 P. M., the lady occupying her usual place at the head of the table. She required no further treatment.

*Comments.* There were present three characteristic symptoms of *Natr. mur.*: Coldness about the heart, blindness and unconsciousness during the chill; and great prostration. Adding to these characteristic symptoms, the irregularity of the pulse, and her exposure to malaria, the choice was very easy; while *Camphor* and *Gelseminum* had some of her symptoms, the totality of them could be found only under *Natr. mur.* Then came to be considered the possibility of a return of the chill in twelve, twenty-four or forty-eight hours, and the great danger to her life from such a recurrance; for she expressed herself as not able to endure such another chill, which surpassed anything she had ever experienced.

Just in such very grave cases does our true healing-art show itself to the greatest advantage, and it never becomes necessary to fly to other means than those offered us inside of our exclusive school of therapeutics. Shall we listen to the seductive voice of public teachers who in such cases claim that it is not only the duty, but the privilege of the homœopathic physician to administer massive doses of *Quinine* to prevent a possibly fatal return of such a chill? We positively decline to listen to such weak teachers! If we are true to Homœopathy it will be true to us—that is our experience; no matter how men may bluster who never tried the experiment. Let them say that a case like this was not cured by the homœopathic remedy faithfully administered; let them say that the curative virtue of medicines is limited to a certain fixed potency; let them call us hide-bound transcendentalists; let them babble about the absurdities of an exclusive dogma and the limits to the applicability of homœopathic practice, all of it will not do away with *facts*, stubborn facts. Such talk only proves the utter incompetency of a set of illogical and ignorant fault-finders, whose only desire is to drag the noble healing-art and the healers down into the detestable mire of eclecticism, which suits them well.

